# Utilizing the Delphi method to develop parent and child surveys to understand exposure to farming hazards and attitudes toward farm safety

**DOI:** 10.3389/fpubh.2022.1027426

**Published:** 2022-12-09

**Authors:** Jessie Adams, Alison Kennedy, Jacquie Cotton, Susan Brumby

**Affiliations:** ^1^School of Medicine, Deakin University, Waurn Ponds, VIC, Australia; ^2^National Centre for Farmer Health, Western District Health Service, Hamilton, VIC, Australia

**Keywords:** Delphi technique, child, parent, risk-taking, agriculture, farms, Australia, attitude

## Abstract

Children on farms are at increased risk of injury. In Australia, children under 15 years consistently represent ~15% of all farm-related fatalities. This study aimed to develop parent and child surveys to gain a greater understanding of children's (5–14 years) exposure to occupational risk on farms by exploring their exposure to farm hazards, risk-taking behavior, their use and attitudes toward safety measures, and experience of farm-related injury. As farming communities are heterogeneous, a modified Delphi method was undertaken to ensure input from a diverse group. Seventeen experts participated in a three round process—the first two rounds required rating of proposed survey questions in an online questionnaire and the final round was an online discussion. Consensus was defined as 75% agreement or higher. This process resulted in 155 parent questions and 124 child questions reaching consensus to include. The modified Delphi method developed surveys that provide insight into the behaviors and attitudes of children (individuals) and their parents on farms (family) and will assist in informing how community, organizations and policy frameworks can improve child safety on farms. It will assist in identifying and understanding common farming exposures/behaviors of children and their parents to inform the development of targeted and culturally appropriate injury prevention strategies. As farming groups are heterogeneous, these survey scan be used on varying farming cohorts to identify their unique farming hazards and challenges. Child farm-related injuries are a problem globally and must be addressed; children are dependent on adults and communities to create safe environments for them.

## Introduction

Globally, children on farms have been identified as vulnerable to injury. In Australia, children represent ~15% of farm-related fatalities; a rate that has remained consistent for over 20 years (1). The key hazards responsible for these deaths are; water bodies, quad bikes, tractors, utility vehicles and cars, motorbikes and horses (1).

While consistent rates of farming injury—and the hazards contributing to injury—are well identified, children's behavior on farms and how they engage with farming hazards is not well understood. Internationally, some research has explored the individual aspects of children's exposure to the farm, including their risk, use of safety measures or farm tasks completed (2–8). Research conducted in Australia has explored fatal injury associated with specific farming hazards, such as water bodies and quad bikes (9, 10). However, much of what is known about behaviors on Australian farms remains anecdotal. To our knowledge there has been no surveys previously developed that sought to investigate children's engagement with the farm, targeting known key hazards, use of safety measures, attitudes toward farm safety and role on the farm.

The farming workplace is frequently also a place of residence and an embedded part of farming family lifestyle, culture and values. As such, children will always be involved in agriculture to some degree. While agriculture remains one of the most dangerous industries in Australia (11), it is imperative to understand how children's engagement can be managed and integrated in the safest way possible.

Shifting culture and patterns of behavior that may have been established over multiple farming generations requires a “whole of community” approach (12, 13). The socio-ecological model (SEM) considers how factors influence individuals' behaviors. Specifically, it highlights the interaction between the individual (intrapersonal), relationships (interpersonal), community and societal factors (14). Understanding how these factors interact and influence the safety of children on farms is important to facilitate behavior, cultural and legislative changes.

Community engaged research has been identified as a tool able to empower communities through inclusion, collaboration and participation. Participatory research can occur on many levels from “inform” where stakeholders are informed on a certain topic, through to “empower” where the community leads the research project (15). There are many benefits to participatory research, including benefits for the community (capacity building and shared decision making) (16); greater relevance and cultural sensitivity of research; greater participant recruitment; and, improved reliability and validity of research outcomes and translation of research findings (17, 18). A valuable tool for community engaged research is the Delphi method (17). The Delphi method was developed by the Research and Development Corporation (RAND) in the 1950s (19, 20). Typically, the multistage technique focuses on gaining consensus from a group of experts on a particular subject (21, 22). While the process has evolved over time, it continues to be based on two fundamental characteristics: (i) a series of iterative rounds where expert panelists provide their opinion, and (ii) the results of each round being shared with panelists before they provide feedback in the next round (23). Based on these fundamental characteristics, the Delphi method allows for flexibility to ensure the process is suitable and appropriate for the aims of the study and the panel of experts recruited (21).

Most research to date that has explored children's engagement with the farm has gathered information from the perspective of the parent. Ehrlich and colleagues (24) matched parent and child surveys on their knowledge, habits and attitudes around safety behaviors, concluding it was inaccurate to rely on parents' responses on their children's use of safety measures, as they overestimated their use. The results also showed a strong association between parents' role-modeling positive safety behaviors and reduced risk-taking by children. This highlighted the need and benefits for injury prevention to be a whole family issue. Therefore, it was deemed important to develop two surveys to gain insight from both parents and children to develop a more holistic understanding of risk-taking behaviors and use of safety measures on farms.

The aim of this study was to utilize a modified Delphi method to develop community-informed parent and child surveys to measure children's exposure to farming hazards, their risk-taking behaviors and experience of farm-related injury, and their use of—and attitudes toward—safety measures. The purpose is to describe a method that can be used by others to develop surveys specific to their country.

## Materials and methods

### The Delphi method: Overview

#### The modified Delphi method used in this study

The Delphi method was used in this study to develop a set of survey questions to explore children's exposure to farming hazards, risk-taking behaviors, use of safety measures and experience of farm-related injury, from the perspective of parents and their children (aged 5–14 years). As such, the following modifications to the traditional process were implemented.

1. Literature review: Traditionally, a single open-ended question would have been asked of panelists in the first round of the Delphi method. In this study, a review of the literature was undertaken to inform the development of a series of proposed closed-ended questions to undergo rating by the panel. This has become a widely accepted modification (25).
2. Rating scale: A five-or nine-point Likert scale is typically used in rating items *via* the Delphi method. In this study, the panel were asked to rate the proposed questions as either “yes” (to be included in the final survey), “no” (to be removed) or “unsure or needs further editing” (26, 27). This meant panelists had to make more definitive decisions with their rating of proposed questions.
3. Anonymity of the panel: Traditionally, Delphi panels remain anonymous to each other throughout the whole process. This is largely to avoid bias and potential influencing on decision-making. However, more recently, the combination of anonymous rounds and a subsequent face-to-face discussion have been successfully used (26, 27). The current three round study consisted of two rounds requiring panelists to complete online surveys (anonymous to other participants) and the final round held as an online discussion. This allowed all panelists to come together to discuss and clarify the remaining questions that were yet to reach consensus after the first two rounds. However, rating of the remaining questions was still completed anonymously.

There were benefits for utilizing a modified Delphi method to develop the two surveys. Previous research has described consensus as a reliable contingency and acceptable to achieve construct validity (28). Hutchings and colleagues (29) determined the Delphi method to be more reliable than nominal groups in using consensus in the development of clinical guidelines. Additionally, the use of the Delphi method allowed a group of experts and end-users from various geographical locations to assist and be involved in the development of the two surveys (30). This resulted in panelists being consulted and involved throughout the development of the surveys ensuring a participatory research process (15). The modest number of participants required allowed the study to be conducted with the limited resources available (31, 32). Utilizing the mixed methods of panelists rating questions on inclusion and providing qualitative responses allowed for thorough feedback and consideration of each proposed question. The online discussion of the remaining questions ensured all panelists could raise their uncertainties. Further, maintaining anonymity of individual panelists rating throughout the process ensured they could be comfortable providing their opinions and not be influenced by others which may have occurred if a focus group was utilized (e.g., dominating personalities or people they may identify as superior) (30). The providing of result reports following the first and second rounds allowed panelists to see how others were rating the proposed questions and the comments provided, this allowed them to reflect and potentially adjust their rating in the following rounds.

Ethics approval was obtained from Deakin University Research Ethics Committee (Ref: 2020-355).

#### Panel selection and recruitment

There are no specific guidelines on who or how many participants to include in a Delphi panel. Each participant must be justified as a topic expert and the panel should represent variation in cognition, expertise and experience (21, 22, 26). Therefore, multidisciplinary experts with varied experience (agriculture, health, research, farming parents, and policy development) from varying geographic locations (across Australia and internationally) were invited to participate.

Potential panelists were identified through a combination of purposive and convenience sampling, drawing on the direct and extended networks of the National Center for Farmer Health (25). These included child farm safety specialists, injury data experts, child farm safety educators, farm safety researchers, farming parents, rural health researchers, agricultural industry and government representatives, and medical professionals. In total, 27 professionals were invited *via* email to participate, resulting in 17 consenting to participate in round one (see Table 1 for description of panel participants).

**Table 1:** Panel participants in the modified Delphi study.

**Participant**	**Location**	**Organization/role**	**Number** ** of rounds completed**
1	Melbourne, Victoria	Agriculture peak body/advocacy group	3
2	Sydney, New South Wales	Government department representing agriculture	3
3	Leongatha, Victoria	Emergency services	3
4	Geelong, Victoria	Farming parent and University researcher	3
5	Kergunyah, Victoria	Farming parent	3
6	Wisconsin, US	Child agricultural health and safety organization	3
7	Melbourne, Victoria	University injury surveillance unit	3
8	Derrinallum, Victoria	Farming parent and child farm safety educator	3
9	British Columbia, Canada	Agricultural health and safety organization (not for profit)	3
10	Hamilton, Victoria	University researcher	3
11	Brisbane, Queensland	Farm safety organization/advocacy group (not for profit)	3
12	Melbourne, Victoria	Child accident prevention organization (not for profit)	3
13	Melbourne, Victoria	Government Department representing health	3
14	Dubbo, New South Wales	University agricultural health and safety center	2
15	Melbourne, Victoria	University accident research center	2
16	Iowa, United States	Agricultural health and safety organization (not for profit)	2
17	Melbourne, Victoria	Workplace health and safety regulator	1

Panel retention has been highlighted as a key aspect of a successful Delphi method (21). The research team encouraged panel retention by: (i) providing a brief description of the overall process so panelists were aware of the total commitment required, (ii) ensuring that those who were invited to participate had a demonstrated interest in the safety of children on farms, (iii) ensuring the process was undertaken in a short time frame, and (iv) encouraging a sense of panelist ownership over the survey development by creating each round from the results of the previous round (21, 26, 33).

#### Consensus

The Delphi method relies on agreement between participants. This “consensus” is defined as the minimum acceptable percentage of agreement between panelists. Literature suggests it is crucial to predefine the level of consensus for a study (21, 26, 33, 34). However, there are no consistent guidelines for determining consensus. Previous literature has suggested varying levels of consensus. Nair et al. (33) recommended between 70 and 80% agreement is appropriate, Niederberger and Spranger (22) endorsed over 60% consensus. Jimenez-Garcia et al. (35), Woodcock et al. (36) and Keeney and colleagues (37) all suggested 75% agreement as appropriate in determining consensus. In the current study, consensus was set at 75% or higher agreement.

#### The three round modified Delphi method

##### Round one: Initial rating of proposed questions

**Figure 1:**
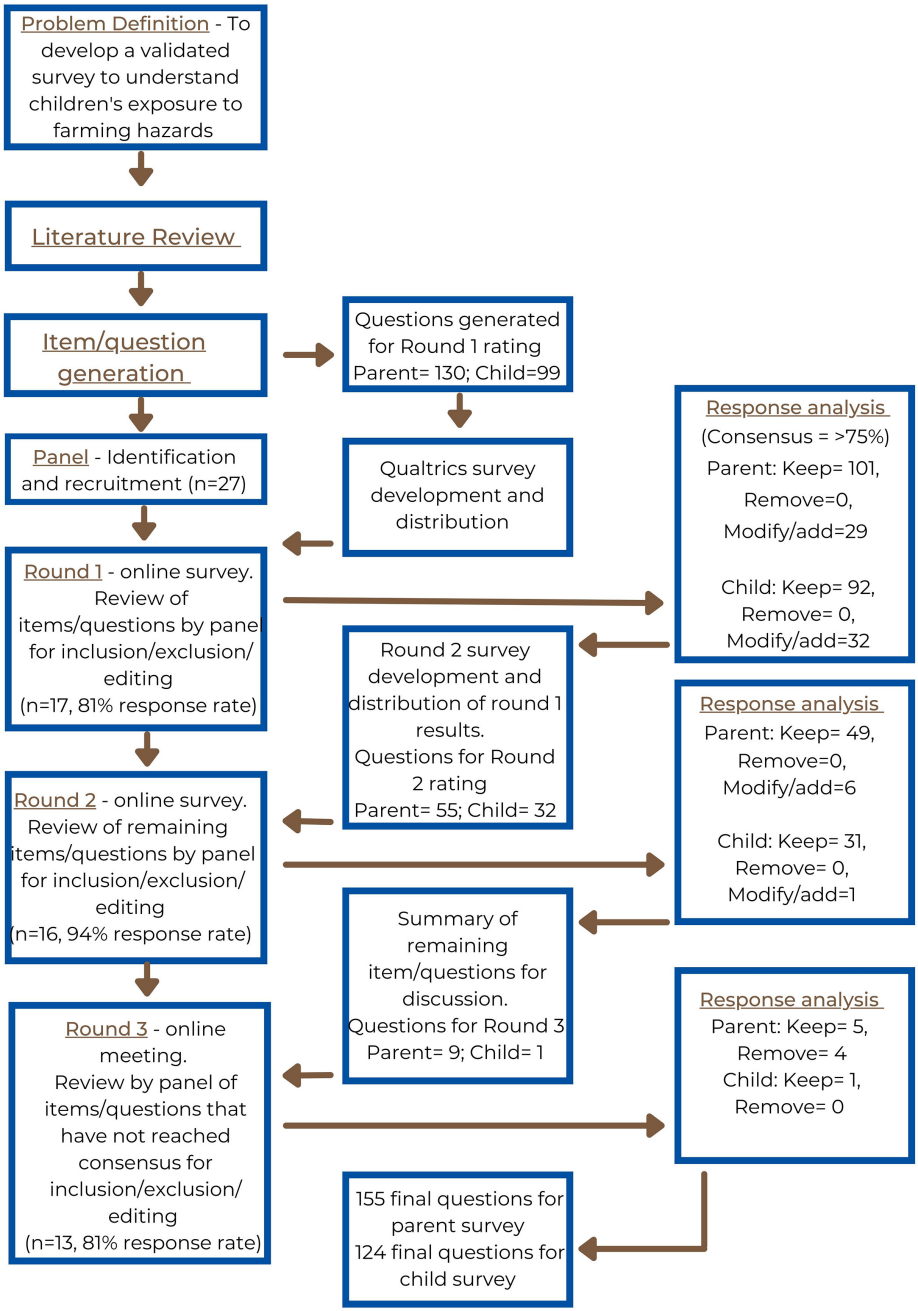
The modified Delphi method utilized in this study (25).

Figure 1 demonstrates the process undertaken in this modified Delphi method. Round one required the 17 panelists to rate proposed questions developed from a review of the literature exploring injury and safety of children on Australian farms (38). The questions developed were a combination constructed by the research team aiming to target the themes and gaps in knowledge identified in the literature review. Where possible, questions that had been used in previous research were utilized (6, 7, 39–45). During question development, all levels of the SEM model were considered including individual (e.g., child demographics); relationships (e.g., the behaviors of those closest to the child including parents); community (e.g., the physical and social environment including the safety measures in place on the farm); and, societal (e.g., cultural norms/influences including what factors influence where children are allowed on the farm) (14). Exploring how these factors interact and influence the safety of children on farms is important to understand behavior and consequently how to influence behavior change.

Questions underwent a pre-Delphi piloting process where a selection of parents (*n* = 7) and their children (within the survey age range of 5–14 years) were asked to provide feedback on question comprehension, wording and overall survey. This ensured the questions presented to the Delphi panel in round one were suitable for the study's target audience. The proposed questions were formatted as an online survey using Qualtrics with questions split into those targeting parents and those for children. Panelists were asked to rate each proposed question using a three-point scale; “yes” (to be included in the final survey), “no” (to be removed), or “unsure or needs further editing”. They were able to provide comments on their decisions, which were then themed and utilized in round two of the process. The three-point scale meant panelists had to make more definitive selections, therefore, assisting in more immediate consensus (33).

Analysis was conducted after each round using the Statistical Package for the Social Sciences (SPSS) (version 27). The percentage agreement on each question was calculated. Agreement of 75% or higher for “yes” or “no” was deemed consensus and the question was removed from future rounds (25, 34, 36). The qualitative comments were grouped per question; the comments on the proposed questions that had not reached consensus to keep were analyzed and alterations were made accordingly. Additionally, new questions were developed from suggestions by the panelists. The altered and new questions were then rated in round two. Result reports were developed for each panel member outlining their individual response per question, the overall panel results and the qualitative feedback (25). Following each round, the result reports were sent to each panelist to allow them to compare their decisions to the overall panel and to see how the survey was being developed from their feedback (21, 22, 30, 33).

##### Round two: Re-rating of proposed questions

Round two followed the same process as the first round with 16 panelists completing the rating of proposed questions. The Qualtrics survey for this round was developed from the results of the first round. This resulted in panelists re-rating questions that had not yet reached consensus (parent survey *n* = 29, child survey *n* = 7) as well as rating additional questions developed from the qualitative feedback provided (parent survey *n* = 26, child survey *n* = 25) (25, 26, 33, 36). In round one, panelists provided qualitative comments after each question category. During analysis, qualitative comments were themed—this included collating comments about each proposed round one question and any additional comments/question suggestions for each question category group. Where deemed appropriate by the research team (i.e., where there was a gap in the proposed questions and where this was considered within the scope of the study), the proposed new questions were then added to the next round of rating, or the wording of previously proposed questions was altered, or multiple choice options were added in previously developed questions. An example of a new question added to round two for rating was “does your child engage with other farming hazards?” This question was developed following panelist feedback on the need to explore other possible hazards. An example of an amended question seeking a more specific understanding was “are there factors you take into consideration when deciding what areas of the farm your child is allowed to go to?” This was amended from “what contributes to your decision to allow your child into the farm workplace?” Rating was completed using the same three-point scale as the previous round. Analysis was conducted in the same way and result reports were disseminated to panelists.

##### Round three: Discussion to reach final conclusions on proposed questions

The final round was held as an online face-to-face Zoom discussion with the aim of reaching efficient consensus on the remaining questions. Until this point, the panel had been anonymous to each other. This modification to the traditional Delphi method has become more common with Boulkedid and colleagues (26) suggesting more than half of all Delphi studies in their systematic review had at least one meeting of panelists. Thirteen panelists attended the online meeting. Each of the remaining questions were presented to the panel (parent survey *n* = 6, child survey *n* = 1) as well as three additional questions for the parent survey, these were all discussed and then rated. The rating of questions was altered slightly from the previous two rounds—panelists were asked to rate questions at the time as either “yes” or “no”—the “unsure” option was removed as panelists could raise their uncertainties and discussion could take place, this was to ensure consensus was achieved by the end of the round. The online program Mentimeter was used for live voting as it ensured rating was still anonymous. If consensus was not reached, further discussion was held and amendments to the question were made before rating was completed again until consensus was reached. Questions that reached consensus to be removed in this round were the disclaimer statements (e.g., “certain authorities” state that children under 16 are not to ride on or operate quad bikes) added in to round two following feedback from some panelists. However, comments provided by other panelists and discussion within the research team highlighted the influence these statements could have on parents answering the survey, resulting in bias. Following discussion in this round, it was agreed that these would be removed.

Following the final Delphi round, the two final surveys were developed in Qualtrics with all questions that had reached consensus to be included. A final review was then conducted by the research team to assess the questions and survey flow/logic, minor alterations and removal of redundant questions was completed.

## Results

### Delphi method for the development of the parent survey

Table 2 demonstrates the results by each round of the Delphi method to develop the parent survey. In the first round, the 17 panelists rated 130 proposed questions, 78% (*n* = 101) of which reached consensus to include in the final survey and 22% (*n* = 29) did not reach agreement. No questions were removed.

**Table 2:** Results of the Delphi method consensus process for the parent survey by round and question category.

**Categories of questions**	**Round one**			**Round two**			**Round three**		
	**Total questions rated by category** ** (*n*)**	Consensus reached to keep^*^ **(*n*)**	**Consensus not reached** ** (*n*)**	**Total questions rated by category^#^** **(*n*)**	Consensus reached to keep^*^ **(*n*)**	**Consensus not reached ** **(*n*)**	**Total questions rated by category^#^** ** (*n*)**	Consensus reached to keep^*^ **(*n*)**	**Consensus achieved to remove** ** (*n*)**
Demographics	14	9	5	4^a^	3	1	2	1	1
General farm safety	5	2	3	4	4	0	1	1	0
General exposure to farming	13	8	5	6	6	0	–	–	–
Safety measures	13	8	5	7	7	0	–	–	–
Water bodies	4	4	0	4	4	0	–	–	–
Quad bikes/side-by-side vehicles	13	12	1	6	5	1	1	0	1
Tractors	6	6	0	8	6	2	2	1	1
Farm vehicles	5	4	1	5	3	2	2	1	1
Motorbikes	6	6	0	2	2	0	–	–	–
Horses	4	4	0	2	2	0	–	–	–
Other hazards	–	–	–	1	1	0	–	–	–
Child role on the farm	15	11	4	2^a^	2	0	–	–	–
Culture/attitudes on child farm safety	10	7	3	2^a^	2	0	1	1	0
Education	–	–	–	2	2	0	–	–	–
Role-modeling	11	11	0	–	–	–	–	–	–
Child farm injury experience	11	9	2	–	–	–	–	–	–
Total	130	101 (77.7%)	29 (22.3%)	55	49 (89.1%)	6 (10.9%)	9	5 (55.6%)	4 (44.4%)

* Consensus was defined as 75% or higher agreement.

# Includes previous round questions where consensus was not reached plus new questions added.

a Questions that were not carried on to the next round were combined with other questions.

The second round consisted of questions that had not reached consensus in the first round, along with additional questions/altered questions following analysis of the qualitative comments. The main themes arising from panelist comments included (i) the need to remove open ended questions and provide multiple choice options, (ii) alterations in question construction and wording, and (iii) the need to focus questions on the key hazards causing child injury.

Of the 55 questions rated, the 16 panelists reached consensus to keep 89% (*n* = 49) of the questions and 11% (*n* = 6) remained unresolved.

The online discussion resulted in 13 panelists coming together to discuss the remaining questions. Five (56%) were kept and four (44%) were removed from the final parent survey.

### Delphi method for the development of the child survey

Table 3 demonstrates the results per round of the Delphi method for the development of the child survey. Panel consensus was very high in the development of the child survey questions. Panelists rated 99 proposed questions for the development of the child survey; 93% (*n* = 92) reached consensus to be included in the final child survey and 7% (*n* = 7) remained unresolved. The qualitative comments provided by panelists on the child survey was consistent with comments on the parent survey. Panelists did highlight some questions were too complex for children so small changes were made to make it simpler for children to answer.

**Table 3:** Results of the Delphi method consensus process for the child survey by round and question category.

**Categories of questions**	**Round one**			**Round two**			**Round three**		
	**Total questions rated by category** ** (*n*)**	Consensus reached to keep^*^(n)	**Consensus not** ** reached ** **(*n*)**	**Total questions rated by category^#^** ** (*n*)**	Consensus reached to keep^*^ ** (*n*)**	**Consensus not** ** reached ** **(*n*)**	**Total questions rated by category^#^** ** (*n*)**	Consensus reached to keep^*^ **(*n*)**	**Consensus achieved to remove ** ** (*n*)**
Demographics	6	6	0	1	1	0	–	–	–
General farm safety	2	2	0	–	–	–	–	–	–
Exposure to farming hazards	8	7	1	3	3	0	–	–	–
Safety measures/farm safety knowledge	15	13	2	2	2	0	–	–	–
Water bodies	5	5	0	1	1	0	–	–	–
Quad bikes/side-by-side vehicles	14	14	0	6	6	0	–	–	–
Tractors	9	8	1	4	4	0	–	–	–
Farm vehicles	7	6	1	5	5	0	–	–	–
Motorbikes	7	7	0	4	4	0	–	–	–
Horses	6	6	0	3	3	0	–	–	–
Other hazards	–	–	–	1	1	0	–	–	–
Child role on the farm	13	11	2	2	1	1	1	1	0
Child farm injury experience	7	7	0	–	–	–	–	–	–
Total	99	92 (92.9%)	7 (7.1%)	32	31 (96.9%)	1 (3.1%)	1	1 (100.0%)	0 (0.0%)

* Consensus was defined as 75% or higher agreement.

# Includes previous round questions where consensus was not reached plus new questions added.

Round 2 saw similar results with 97% (*n* = 31) reaching consensus to include and 3% (*n* = 1) remained. The panel reached consensus to keep the remaining proposed question during the online discussion round.

Table 4 provides a summary of results of the Delphi method showing 155 questions reached consensus to be included in the parent survey and 124 for the child survey.

**Table 4:** Summary table of the Delphi method for each round.

	**Parent survey *n* (%)**	**Child survey *n* (%)**
**Round one**		
Total questions in round	130	99
Consensus to keep	101 (77.7)	92 (92.9)
Consensus not achieved	29 (22.3)	7 (7.1)
**Round two**		
Total questions in round	55	32
Consensus to keep	49 (89.1)	31 (96.9)
Consensus not achieved	6 (10.9)	1 (3.1)
**Round three**		
Total questions in round	9	1
Consensus to keep	5 (55.6)	1 (100.0)
Consensus not achieved	4 (44.4)	0 (0.0)
**Total questions reached consensus to include in final surveys**	155	124

## Discussion

The modified Delphi method utilized in this study was effective in developing two surveys to explore children's exposure to farming hazards, risk-taking behaviors, attitudes and use of safety measures, and experience of injury. The mixed method nature of the modified Delphi (including rating and qualitative data) allowed for a more thorough identification of appropriate questions, as well as a greater understanding of panelist reasoning behind the rating choices and the subsequent amending or adding new questions. Furthermore, holding the final (3rd) round as a live online discussion with anonymous ratings, encouraged varying opinions to be shared and allowed all panelists to feel comfortable providing their individual opinions.

The results of the Delphi method highlighted the value and importance of collaboration and participatory research. Although there was >70% agreement in the parent and 90% in the child proposed questions after the first round of rating, uncertainty still remained in 20% and 7% of proposed questions, respectively. This collaborative/participatory method enabled improvements in the relevance and cultural sensitivity of the surveys (17, 18). The comments and level of disagreement on some proposed questions demonstrates the process rigor. This resulted in improved use of language, additional collaboratively-agreed-upon questions, inclusion of appropriate examples and multiple choice options. While efforts were made to ensure a diverse representation of experts involved in the Delphi, the overall number of participants (*n* = 17) was not large. Consideration for inclusion in the panel included organizational affiliation, experience, academic qualification, geographical location and recommendation by others (22, 31). In a Delphi method, group size does not rely on statistical power and there is no agreed upon minimum number of panelists recommended (31). Rather, the focus is on multidisciplinary representation, differences in cognition, expertise and experiences as well as potential group dynamics (22, 26, 46).

A potential limitation to this study was the high consensus between the panel members in regard to the child survey. It is suggested this may be due to the child survey questions being presented after the adult questions in every round (47). It is not believed this influenced the development of validity of the final child survey as the additional questions introduced in round two were aligned to the results of the parent survey to ensure the two surveys would be comparable. It is recommended in future research that the order of the proposed questions for a survey be changed between rounds, as panelists may fatigue toward the end of each round.

The Delphi method relies on group consensus, and while the panelists that participated in this study were deemed “experts” in the field, there is the potential that the results are not necessarily the most correct, as they are still based on opinion (23). However, when there is no other evidence available, the reliance on group opinion is believed to be a better basis and superior than the dependence on an individual judgement (48, 49). As child farm-related injury rates have remained consistent over an extended period, a new approach is required.

The consensus between panelists to “keep” questions was high, resulting in a large number of questions to be included in the final surveys. As described above, following the completion of the three Delphi rounds, the research team assessed the questions and removed any questions that had become redundant throughout the rounds. Further, survey logic was added to ensure participants were not asked to answer any questions that were not relevant to them, reducing completion time.

Following the development of the two surveys, the parent survey will be promoted throughout rural/regional Victoria for completion. Once a parent completes the survey, they will be emailed a link for their child to complete the survey. It is aimed 100 surveys will be completed in this study.

## Conclusion

The survey age range (5–14 years) will facilitate a greater understanding of the different safety attitudes and farm activities that children—of varying ages—are undertaking on farms. Previous international research has described children's engagement on the farm as varying—depending on factors such as age and developmental level (3, 4, 8). Future analysis of survey results, will enable comparisons between different age cohorts to see trends in children's behaviors on the farm as they develop. This should assist in the identification and improvement of age appropriate interventions.

This modified Delphi method supported the development of surveys that can assess the behaviors and attitudes of children (individuals), and their parents (relationships) on farms. This will provide insight on how community, organizations and policy frameworks can interact to assist in the development of effective and appropriate interventions to improve child safety on farms. As farming communities are heterogeneous, these surveys will be able to be used across varying farming cohorts (e.g., geographic or industry) to identify specific challenges/behaviors, and assist in developing targeted and appropriate responses to child safety. The resulting surveys may be used longitudinally—with the ability to track change in industry behaviors and attitudes overtime and evaluate the effect of any interventions and parental awareness of safety for children on farms.

Child farm injury and fatalities have been consistent and an ongoing global shame for centuries. Children are reliant on the adults around them to provide them with a safe environment. Therefore, more needs to be done to understand the farming life/behaviors of children. This study utilized a modified Delphi method that resulted in the development of parent and child surveys to explore children's exposure to farming hazards, risk-taking behaviors, attitudes and use of safety measures and experience of farm-related injury. The consideration of each of the SEM levels within this study will ensure factors influencing behaviors are identified to assist in developing effective, appropriate and targeted “whole of community” initiatives to address child farm-related injury.

## Data availability statement

The raw data supporting the conclusions of this article will be made available by the authors, without undue reservation.

## Ethics statement

The studies involving human participants were reviewed and approved by Deakin University Research Ethics Committee (Ref: 2020-355). The patients/participants provided their written informed consent to participate in this study.

## Author contributions

JA conducted the data collection, data analysis, and drafting of the manuscript. AK, SB, and JC provided guidance and supervision throughout the study. All authors contributed to the study design, reviewing and editing of the manuscript, and approved the final manuscript.
